# Biochemical and histological alterations induced by nickel oxide nanoparticles in the ground beetle *Blaps polychresta* (Forskl, 1775) (Coleoptera: Tenebrionidae)

**DOI:** 10.1371/journal.pone.0255623

**Published:** 2021-09-24

**Authors:** Saeed El-Ashram, Awatef M. Ali, Salah E. Osman, Shujian Huang, Amal M. Shouman, Dalia A. Kheirallah

**Affiliations:** 1 College of Life Science and Engineering, Foshan University, Foshan, Guangdong, China; 2 Faculty of Science, Kafrelsheikh University, Kafr El-Sheikh, Egypt; 3 Department of Zoology, Faculty of Science, Alexandria University, Alexandria, Egypt; Beni Suef University Faculty of Veterinary Medicine, EGYPT

## Abstract

The present study evaluates the effect of nickel oxide nanoparticles on some biochemical parameters and midgut tissues in the ground beetle *Blaps polychresta* as an indicator organism for nanotoxicity. Serial doses of the NiO-NPs colloid (0.01, 0.02, 0.03, 0.04, 0.05, and 0.06 mg/g) were prepared for injecting into the adult beetles. Insect survival was reported daily for 30 days, and the sublethal dose of 0.02 mg/g NiO-NPs was selected for the tested parameters. After the treatment, nickel was detected in the midgut tissues by X-ray microanalysis. The treated group demonstrated a significant increase in aspartate aminotransferase (AST) and alanine aminotransferase (ALT) activities when compared to the untreated group. However, the treated group demonstrated a significant decrease in ascorbate peroxidase (APOX) activity when compared to the untreated group. Histological and ultrastructural changes in the midgut tissues of treated and untreated beetles were also observed. The current findings provide a precedent for describing the physiological and histological changes caused by NiO-NPs in the ground beetle *B*. *polychresta*.

## 1. Introduction

The production of nanoparticles has increased due to the instant progress of nanotechnology [1]. With the progress of nanotechnology, metal oxide nanoparticles (MONPs) have been widely used in different fields, for instant paints, cosmetics, electronic devices, additives in food, and medical and biological systems [2, 3]. With the excessive use of MONPs, studies have investigated their adverse effects on the environment, human health, and soil organisms [4]. Derived nanoparticles (NPs) can induce different interactions in living organisms [5].

The size of NPs enables them to reach the cell‘s nucleus through the nuclear pore and interact directly with the DNA in the chromosomes, causing genetic damage. Also, they can interact with proteins involved in DNA replication and generate high quantities of oxidative stress that induces DNA damage [6].

Nickel (Ni) is combined with some metals to make alloys, such as stainless steel [7]. It is extensively used in industry due to its toughness, hardiness, high resistance to corrosion and rusting, and it also has good plasticity [8]. Industrial activities can raise the Ni concentration in the environment [8]. Ni has low solubility in water, which indicates its toxicity effects [9]. Exposure of organisms to Ni or its compounds induces different pathological effects, such as inflammation, allergy reactions, teratogenicity in the human body, lung fibrosis and lung cancer [8]. Nickle nanoparticles (Ni-NPs) are used in biological medicine and may induce cardiac toxicity, liver and spleen injury, and lung inflammation [8].

Insects have the ability to act as environmental monitors in a variety of situations [10]. In addition, insects have a short generation period, are very prolific, are inexpensive, and offer good genetic tools for studying human-related illnesses, such as cancer and tumors [11, 12]. Beetle genetic sequences are accessible, and parts, including the midgut, reproductive organs, and fat bodies, are quite straightforward to access for study [13]. Tenebrionid beetles are excellent ecological models since they live in a variety of environments [14]. Their behavior is inextricably linked to human activities, like urbanization, agricultural areas, and plantations [15]. They’re also incredibly adaptable to harsh climatic circumstances, and, unlike other insects, they live for a long time and maintain a steady population [16]. The ground beetle B. polychresta (Coleoptera: Tenebrionidae) is the most common tenebrionid and may be seen in enormous numbers [17]. The toxicity of nanoparticles has been studied using zebrafish [18]. Due to its fast embryonic growth, it shows complex behaviors and may be utilized as an animal model under particular physical circumstances (temperature ≤ 28°C). However, since it needs a low temperature, it might be challenging to utilize as an ecological model at times. As a consequence, the data may be erroneous [19].

Biochemical reactions are the result of an organism’s response to a stressor [20]. Biochemical analysis is progressively used in environmental risk assessments to monitor the prevalence of xenobiotics [21]. Therefore, biochemical alterations demonstrate the negative effect that results from vulnerability to a contaminant [22]. The detoxification enzymes in insects are the defence barriers against foreign compounds and they have significant roles in preserving normal physiological functions [23]. Aspartate aminotransferase (AST) and alanine aminotransferase (ALT) aid energy production and serve as a connection between the protein and carbohydrate metabolism [24]. These enzymes are known to be changed during various pathological conditions. The increase in transaminases is considered evidence of cellular leakage and loss of functional cell membrane integrity [23].

Reactive oxygen species (ROS) can damage proteins, lipids, and other important macromolecules in the insect’s body. Therefore, organisms must scavenge ROS before cellular damage [25]. Ascorbate peroxidase (APOX) reduces H_2_O_2_ with concordant ascorbate oxidation [26]. Insects distinctly lack glutathione peroxidase and, since catalase has a poor affinity for H_2_O_2_, the APOX enzyme may have a significant role in removing H_2_O_2_ in insects [27].

The midgut epithelium is the principle site that manages the detoxification of ingested xenobiotics [28]. It is considered an important organ for toxicity analysis because the accumulation of metals occurs in the midgut [29]. Moreover, it is one of the primary interfaces where insects come in contact with injected metals [30]. MO-NPs are capable of inducing cellular and subcellular modifications in the midgut epithelium in insects [31]. Therefore, histological and ultrastructure inspections help in monitoring the pathological effects even at the sublethal level [32].

The current study aims to assess the biochemical, histological, and ultrastructure changes that resulted in the ground beetle *B*. *polychresta* as a sensitive indicator organism for nickel oxide nanoparticles (NiO-NPs) that may be accumulated in the environment by industrial activities.

## 2. Materials and methods

Ethics Statement: The ethical rules for animal regulations were followed and approved by the Faculty of Science, Alexandria University committee in January 2014(Alex-11-2014). All institutional and national Guidelines for the care and use of animals (insects) were followed.

### 2.1. The studied insect

According to Condamine *et al*. [33], beetles were identified as *Blaps polychresta* in the family Tenebrionidae at the Faculty of Agriculture, Alexandria University, Entomology Department.

### 2.2. Sampling procedure

One-hundred and forty beetles were sampled from a non-contaminated site, the garden of the Faculty of Science, Elshatby, Alexandria University, Alexandria, Egypt [34].

To guarantee that the area would not be affected, the beetle collection site was situated on private property. They were preserved alive in local soil and plants in glass cages in the laboratory. They were kept at a temperature of 29 ± 3°C and humidity of 85% RH, similar to their place of origin.

The beetles were divided into seven groups, one untreated group and six treated groups with different nickel oxide nanoparticles (NiO-NPs) concentrations. Each group contained 20 adult beetles.

### 2.3. Synthesis of nickel oxide

Nickel (II) oxide (NiO) nanopowder [Product No.: 637130, APS: <50 nm (BET) with Purity: 99.8% trace metal basis], was purchased from Sigma-Aldrich Co. Ltd, St. Louis, MO, U.S.A. Particle size and morphology were characterized by a Transmission Electron Microscope (JEOL, JEM-1400 plus Electron Microscope).

The NiO-NPs colloid was prepared as follows: 1. Particles were suspended in normal saline (0.6%) with a final concentration of stock solution of 0.1 mg/ml, 2. sonication for 1 min using a Branson sonifier 450 (Branson Ultrasonics Corp., Danbury, CT, U.S.A.), 3. the suspension was kept on ice for 15 sec, 4. sonication on ice for 10 min at a power of 400 W. Five serially diluted doses were taken from the stock solution (0.01, 0.02, 0.03, 0.04, 0.05, and 0.06 mg/g). All samples were prepared under sterilised conditions.

### 2.4. Method of treatment

Beetles were injected with serial doses of 0.01, 0.02, 0.03, 0.04, 0.05, and 0.06 mg/g from the NiO-NPs colloid for the determination of the median lethal dose (LD_50_), which is the dose required to cause 50% mortality. The untreated group received an injection of normal saline. The weight of the adult beetle was approximately 1.87 g.

Beetles were injected while still alive in the ventro-caudal between the 4th and 5th abdominal sclerites. Each beetle was placed on its ventral side and injected with a 1 ml BD hypodermic syringe (27G, "1/ needle) filled with different doses of the NiO-NPs colloid [35] (S2 Fig). The needle was kept horizontally as possible and only its tip was injected to prevent any physical damage of the internal tissues [36]. Mortality was reported daily for 30 days. Cumulative mortality was calculated for each tested dose. The LD_50_ of NiO-NPs was determined by the log-probit model using the LdP LineR software (Ehabsoft (http://www.ehabsoft.com/ldpline)) (Table 1, S3 Fig).

**Table 1:** Dose-response percentages of NiO-NPs in the studied groups.

Dose	Dose 1000 00	Log (Dose1000000)	Treated	Observed mortality response %	Linear mortality response %	Linear probit
0.000001	1	0.0000	20	5.000	2.14291	2.9710
0.01	10000	4.0000	20	35.000	46.6310	4.9154
0.02	20000	4.3010	20	35.000	52.4605	5.0618
0.03	30000	4.4771	20	45.000	55.8561	5.1474
0.04	40000	4.6021	20	50.000	58.2429	5.2081
0.05	50000	4.6990	20	70.000	60.0742	5.2552
0.06	60000	4.7782	20	95.000	61.5507	5.2937

In the mortality test (LD_50_), the sublethal dose was 0.02 mg/g. The group that received this dose was considered as the treated group (group 2). The tested parameters (enzyme activities, histological analysis, and ultrastructure analysis) were investigated in this group [31].

### 2.5. Nickel X-ray detection in midgut tissues of *B*. *polychresta*

The Ni percentage was determined in midgut tissues by using an energy-dispersive X-ray microanalysis (JEOL (JSM-5300) scanning microscope at the Electron Microscope Unit, Faculty of Science, Alexandria University, Egypt). Eight samples of midgut tissues were analysed from each group to estimate the accuracy of the analytical results. SEM EDX software was used to identify the peaks for each metal in the tissue. For each element in the sample, line intensities were measured. It was also measured for the same elements in calibration standards of known composition. A stationary spot (X500) was analysed at random for 110 s.

### 2.6. Determination of aspartate amino-transferase (AST), alanine amino-transferase (ALT) activities in midgut tissues

Levels of the enzymes AST and ALT in midgut tissues were estimated colorimetrically using a kit purchased from Quimica Clinica Aplicada S.A. Co., Spain, according to the method of Reitman & Frankel (1957) [37].

### 2.7. Determination of ascorbate levels (APOX) in midgut tissues

Eight samples of the beetles’ midgut tissues were homogenized and centrifuged (8000 g, 5 min), and kept on ice. Samples were mixed with 2 M Tris buffer (pH 9.2, 26% v/v) and analysed immediately with reverse-phase HPLC [38]. Ascorbate was separated with a Vydac C-18 column (201 HS, 250 X 4: 6 mm) and guard column, using a mobile phase composed of aqueous ammonium phosphate (20 mM) and EDTA (1.0 mM). The flow rate was 1.0 ml/min (35°C). The peak area was measured with a Shimadzu UV–visible detector (265 nm), and the peak area was integrated with a Shimadzu C-R4A integrator. The identity of the ascorbate peak was determined by analysis of ascorbate standards and confirmed by the treatment of standards and samples with ascorbate oxidase (10 EU (enzyme units)/ml neutralized sample). Peak areas were converted to mU/mg protein injected using an ascorbate standard curve.

### 2.8. Histological and ultrastructure preparations

The midguts were fixed in _4_F_1_G in phosphate buffer solution (pH 7.2) at 4°C for 3 h and post-fixed in 2% OsO_4_ in the same buffer for 2 h. Samples were washed in the buffer and dehydrated at 4°C through a series of ethanol. Specimens were immersed in an Epon-Araldite mixture in labelled beam capsules. The Ultramicrotome (LKB; Bromma-2088-Ultratome®V, Sweden) was used for the semithin sections (1 μm thick). Sections were mounted on a glass slide, stained with toluidine blue and examined with a light microscope to determine the orientation and the structural features. Photomicrographs were taken at different magnifications by the Olympus CX31, Leica Ultra Cut R light microscope.

Ultrathin sections (0.06–0.07 μm thick) were cut for transmission electron microscope (TEM) then picked upon 200 mesh naked copper grids. Grids were stained for 30 min with uranyl acetate and 20–30 min with lead citrate (Reynolds 1963). Several magnifications were taken for the electron micrographs. Photographing and scoping the grids were achieved by Jeol 100 CX Tem, at the E.M. Unit, Faculty of Science, Alexandria University, Egypt (JEM-1400 plus; JEOL Ltd., Akishima, Tokyo, Japan)

### 2.9. Data analysis

The log-probit model, LdP Line^R^ software (Ehabsoft (http://www.ehabsoft.com/ldpline)) was used to estimate the LD_50_. To estimate mortality, the Kruskal Wallis test for abnormally distributed quantitative variables and comparisons between more than two studied groups was used, and post-hoc (Dunn’s multiple comparisons test) for pairwise comparisons. The IBM SPSS software package version 20.0 program (IBM Corp., Armonk, New York, U.S.A.) was used for the analysis of the X-ray and enzymes [39]. The Shapiro-Wilk test was used to prove the normality of the distribution of variables. The Student *t*-test was used to ascertain the difference between the two studied groups for normally distributed quantitative variables [40]. The significance of the results was judged at *P* ≤ 0.05.

## 3. Results

### 3.1. NiO-NPs characterizations

**Fig 1:**
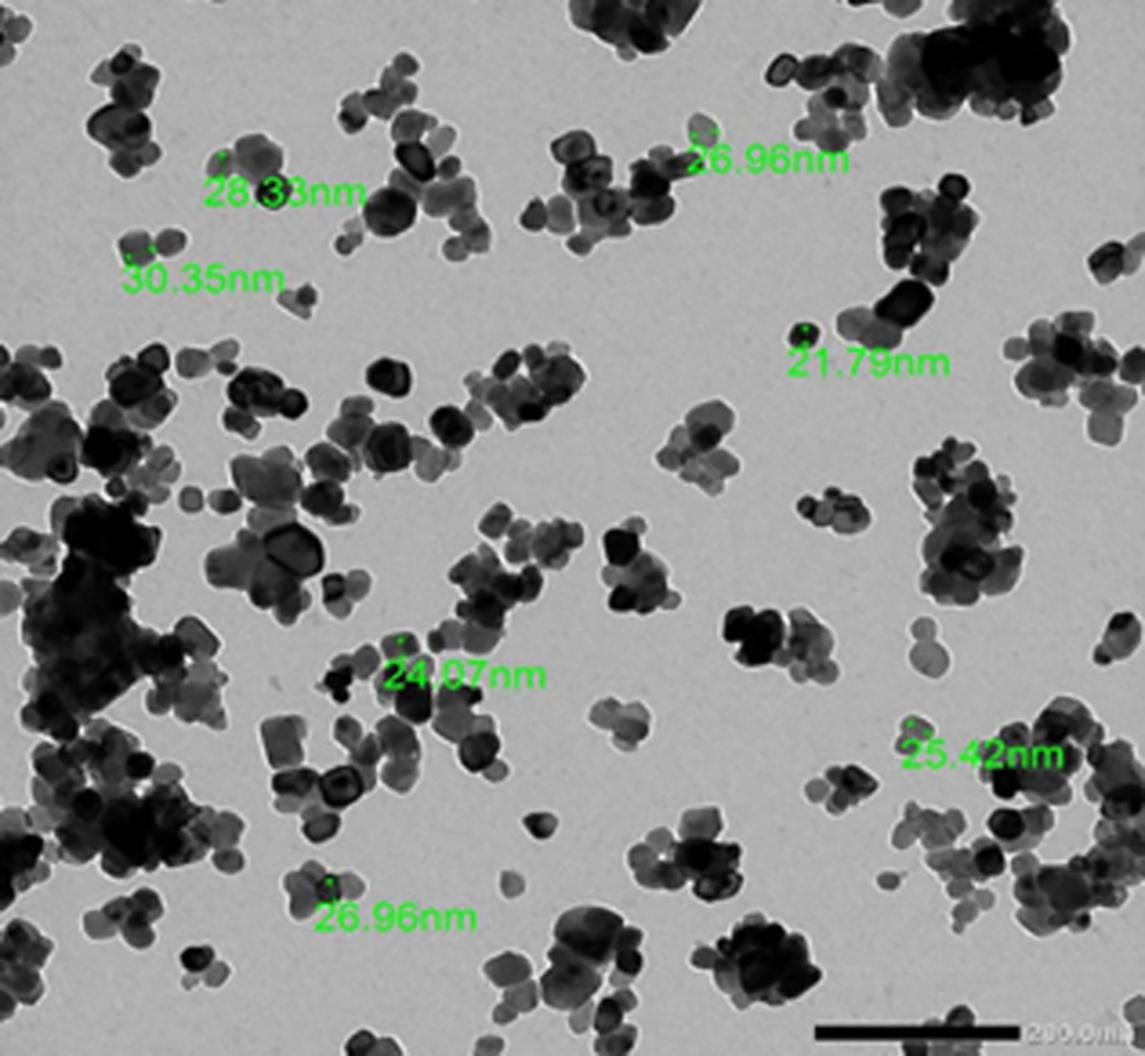
Transmission electron micrograph of NiO-NPs.

A Transmission Electron Microscope (TEM) was used to determine the physical characteristics of NiO-NPs. The micrographs showed that the particles were spherical or oval and aggregated together. The size of the particles ranged from 21.79 to 30.35 nm diameter with a mean diameter of 26.27 ± 4.43 nm which was similar to the manufacture’s references (<50 nm) (Fig 1).

### 3.2. Insect mortality

**Fig 2:**
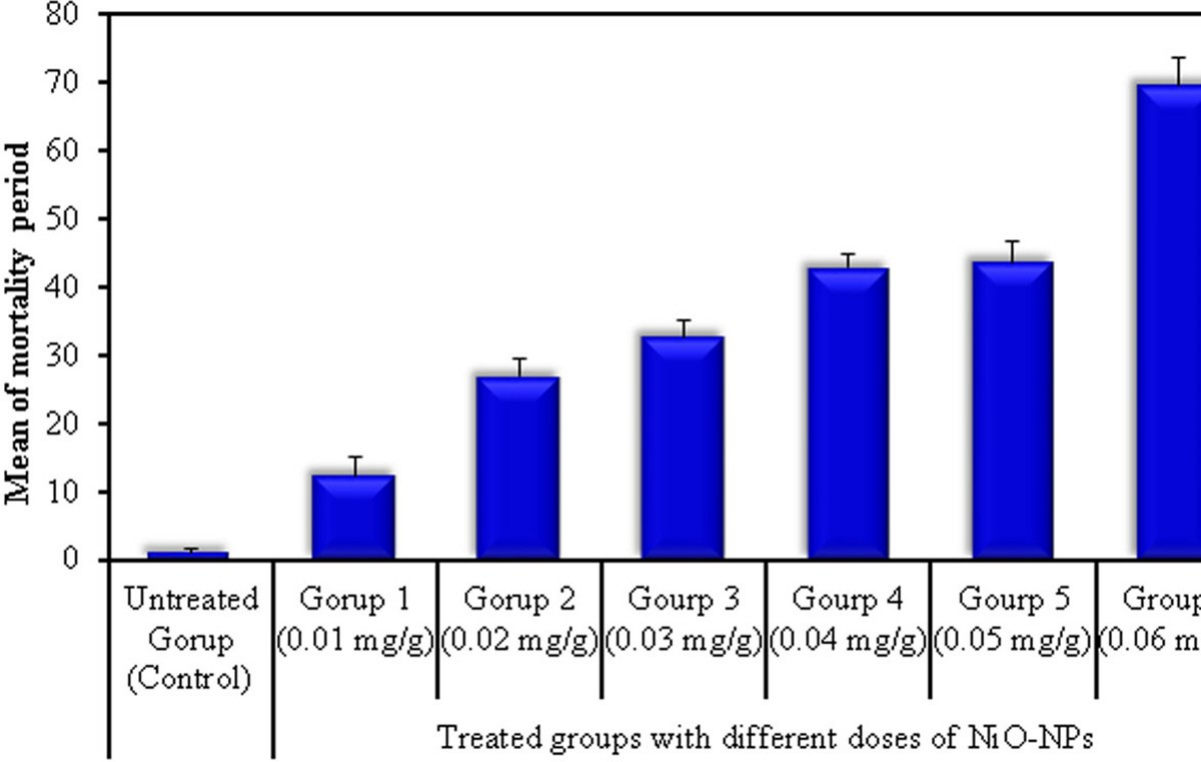
Cumulative mortality percentage in the studied groups during the studied period. The data is presented as a mean±SE.

Beetle mortality was calculated from day 1 to day 30 (S1 & S2 Tables). On day 30, it was observed that 13 beetles in groups 1 and 2, 11 beetles in group 3, 10 beetles in group 4, and 6 beetles in group 5 were alive with the injection of 0.01, 0.02, 0.03, 0.04, and 0.05 mg/g respectively, although, total mortality was observed in group 6 (0.06 mg/g) (S1 Table). It was noticed that the 0.04 mg/g dose resulted in 50% mortality and the 0.06 mg/g dose resulted in 100% mortality. A significant difference in the cumulative mortality percentages between the tested groups was observed. (Fig 2, S2 Table). From Table 1, the LD_50_ was reported at 0.04 mg/g dose, so all the tested parameters were performed in the group that was treated with the sublethal dose of 0.02 mg/g and compared with the untreated group.

### 3.3. Nickel X-ray detection in the midgut of *B*. *polychresta*

Ni was detected in the midgut tissues of the treated group by X-ray analysis to determine the metal percentages (Table 2, S4 Fig). Eight elements were detected in the midgut tissue of the untreated group (Na, Al, P, S, K, Ca, Cu, and Zn), whereas, nine elements (Na, Al, P, S, K, Ca, Cu, Zn, and Ni) were detected in the midgut tissues of the NPs treated group, which indicates that Ni was present in the midgut tissues due to the treatment.

**Table 2:** Trace metal percentages (%) in midgut tissues of *B*. *polycresta* of the studied groups.

Metals	Untreated group	Treated group (Group 2)	*t*	*P*
Na	5.63 ± 0.48	5.93 ± 1.12	0.246	0.814
Al	10.40 ± 2.10	9.73 ± 3.86	0.154	0.883
P	12.38 ± 2.86	25.20 ± 7.28	1.640	0.178
S	42.65 ± 1.82	29.50 ± 5.25	2.369	0.082
K	4.63 ± 1.33	7.83 ± 3.05	0.963	0.389
Ca	3.78 ± 1.44	2.03 ± 0.53	1.142	0.297
Cu	5.33 ± 0.36	5.55 ± 0.56	0.337	0.748
Zn	4.18 ± 0.36	3.68 ± 0.42	0.904	0.401
Ni	ND	3.45 ± 0.36	–	–

For each metal, the percentage expressed by using minimum–maximum values and mean (n = 8) using Student t-test, *statistically significant at *P* ≤ 0.05; ND: not detected.

### 3.4. Determination of aspartate amino-transferase (AST), alanine aminotransferase (ALT) and ascorbate peroxidase (APOX) activities in midgut tissues of beetles in the studied groups

**Fig 3:**
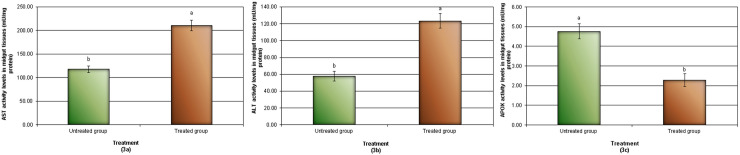
Activities of antioxidant enzymes a: AST, b: ALT, c: APOX in the studied groups. Data are expressed as mean± SE.

In the treated community, AST and ALT enzyme activity levels were significantly higher than in the untreated group in the midgut tissues of beetles (Fig 3A and 3B). However, a substantial reduction in the level of APOX in midgut tissues was found in the treated group as opposed to the untreated group (Fig 3C).

### 3.5. The external morphology of the alimentary canal of *B*. *polychresta*

**Fig 4:**
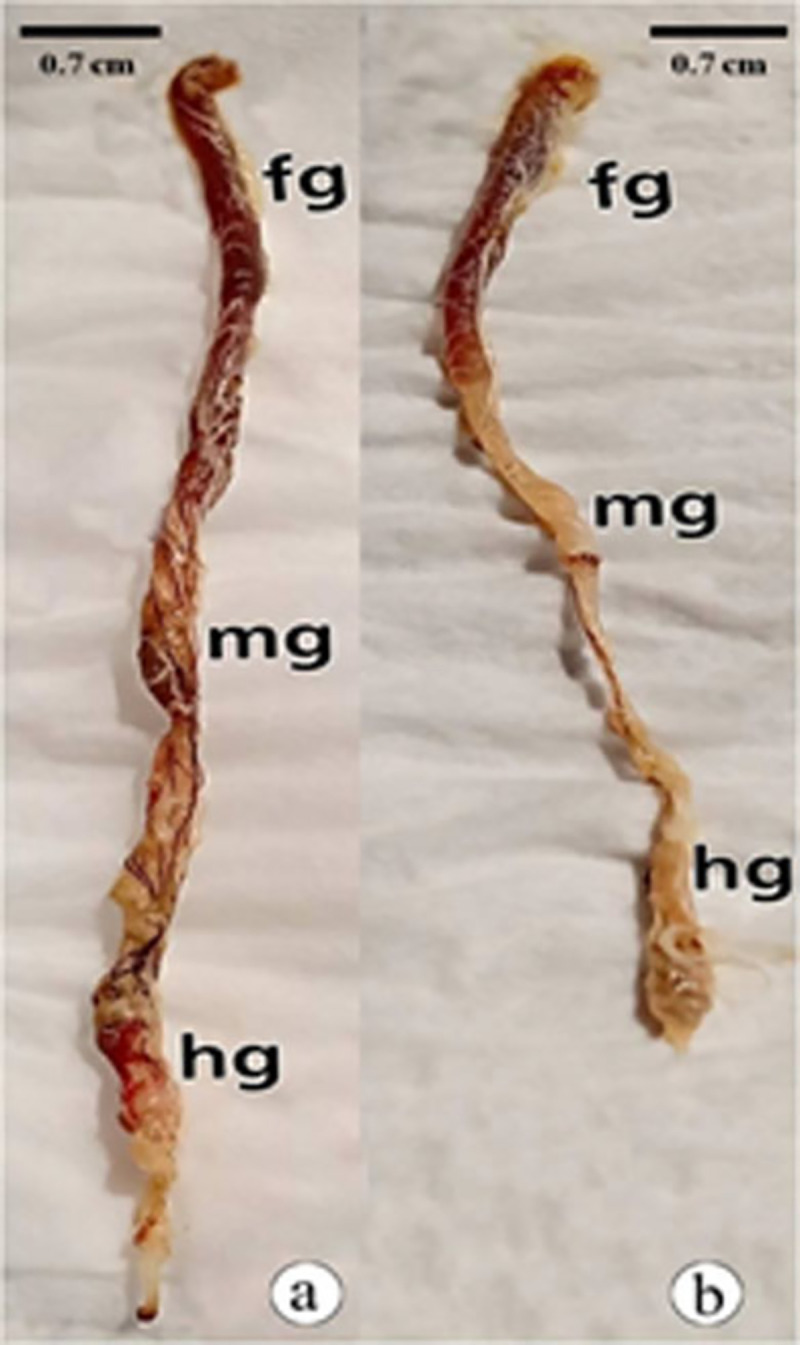
Photograph of the alimentary canal of *B*. *polychresta*, a: Untreated group b: Treated group. fg: foregut, mg: midgut, hg: hindgut.

The alimentary canal of adult *B*. *polychresta* composed of a short foregut, long midgut, and hindgut (ileum, colon, rectum, and anal canal), which opens outside through the anus between the 8th and 9th sternites (Fig 4A). The anatomical deformity that was observed in the alimentary canals of the treated group was the reduction in the length of the canal, being 7.80 cm in the untreated group (Fig 4A) and 5.60 cm in the treated group (Fig 4B).

### 3.6. Histological observations of midgut tissues of adult *B*. *polychresta* in the untreated group

**Fig 5:**
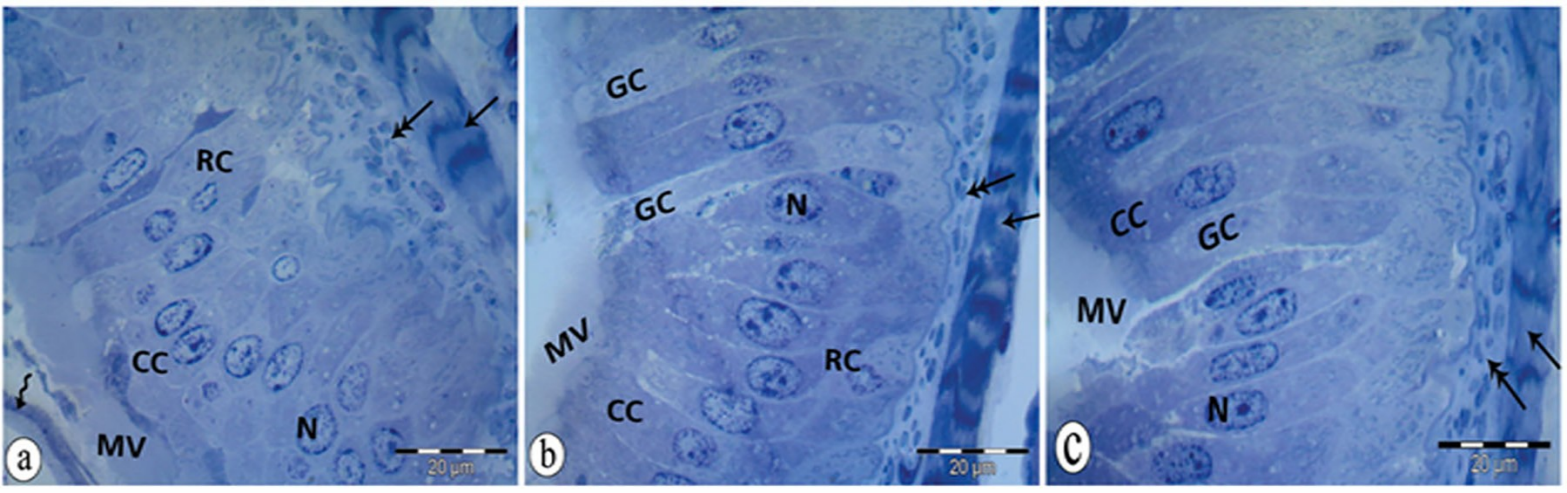
A semithin sections of the midgut epithelium a: circular muscle (arrow), longitudinal muscle (double head arrow), regenerative cell (RC), columnar cell (CC) nucleus (N), microvilli (MV), peritrophic membrane (wavy arrow). b: circular muscle (arrow), longitudinal muscle (double head arrow), regenerative cell (RC), columnar cell (CC), nucleus (N), goblet cell (GC), microvilli (MV). c: circular muscle with light and dark bands (arrow), longitudinal muscle (double head arrow), columnar cell (CC), nucleus (N), goblet cell (GC), microvilli (MV).

The histological structure of the midgut revealed an epithelium lying on a basement membrane coated by two muscle layers, the circular and the longitudinal muscle fibres (Fig 5A–5C). The epithelium consisted of regenerative cells and digestive cells; columnar cells and goblet cells. Each of these cells had a large nucleus and basophilic cytoplasm (Fig 5A–5C). The regenerative cells appeared singly or in clusters called “nidi”, forming crypts near to the muscle fibres (Fig 5A). In the basal lamina, columnar and goblet cells were observed (Fig 5A–5C). The basal lamina of the epithelium was provided with a striated brush border called microvilli (Fig 5A–5C) that expanded the absorption of the cell surface. The peritrophic membrane appeared visible with multilayer and normal thickness. It was separated from the epithelial cells (Fig 5A).

### 3.7. Histological observations of midgut tissues of adult *B*. *polychresta* in the treated group

**Fig 6:**
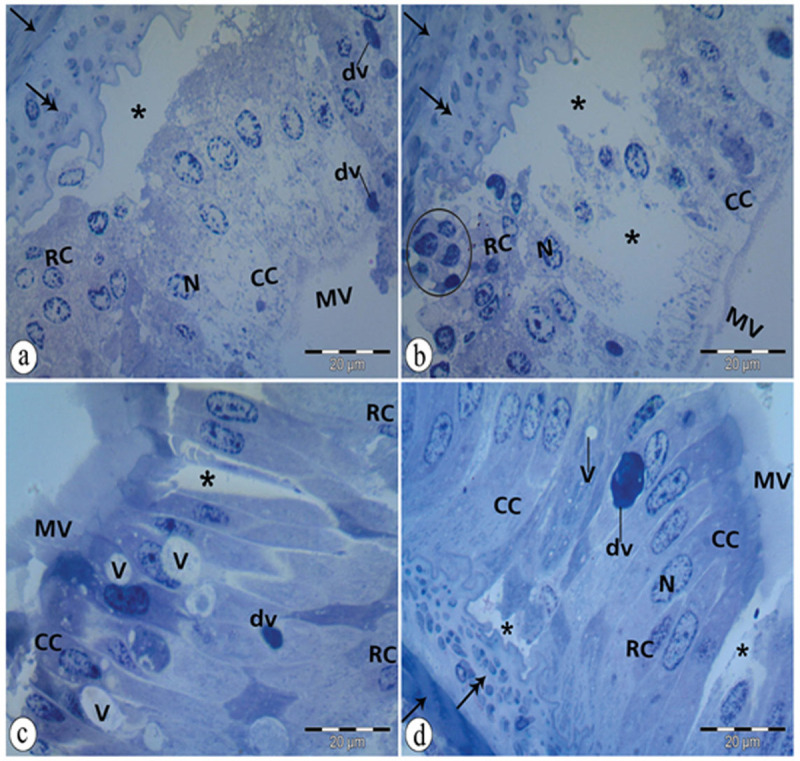
A semithin section of abnormal midgut epithelium. a: lytic cytoplasm (*), regenerative cell (RC) with pale nucleus (N), distorted columnar cell (CC), ruptured microvilli (MV), circular muscle (arrow), longitudinal muscle (double head arrow), dense vesicle (dv). b: massive disruption of the midgut epithelium with lytic cytoplasm (*), distorted microvilli (MV), necrotic regenerative cells (RC) with pyknotic nuclei (circle), columnar cell (CC) with pyknotic nuclei. N: nucleus, double head arrow: longitudinal muscle. c: lytic cytoplasm (*), distortion of microvilli (MV), columnar cell (CC) with a heterochromatic nucleus, regenerative cell (RC), vacuoles (V), dense vesicle (dv). d: lytic cytoplasm (*), microvilli (MV), regenerative cell (RC), columnar cell (CC), nucleus (N), vacuole (V), dense vesicle (dv), circular muscle (arrow), longitudinal muscle (double head arrow).

Histological alterations in the midgut structure were observed in the treated beetles. Numerous vacuoles, lytic areas, and dense vesicles in the cytoplasm of both cells were observed (Fig 6A–6D). Necrotic regenerative cells with pyknotic nuclei as well as a columnar cell with pyknotic nuclei were distinguished (Fig 6B). Also, disruption of microvilli was observed (Fig 6A–6C). The peritrophic membrane was not visible in the micrographs. It might be affected by the NiO-NPs treatment (compare Figs 5A with 6B).

### 3.8. Ultrastructure observations of midgut tissues of adult *B*. *polychresta* in the untreated group

**Fig 7:**
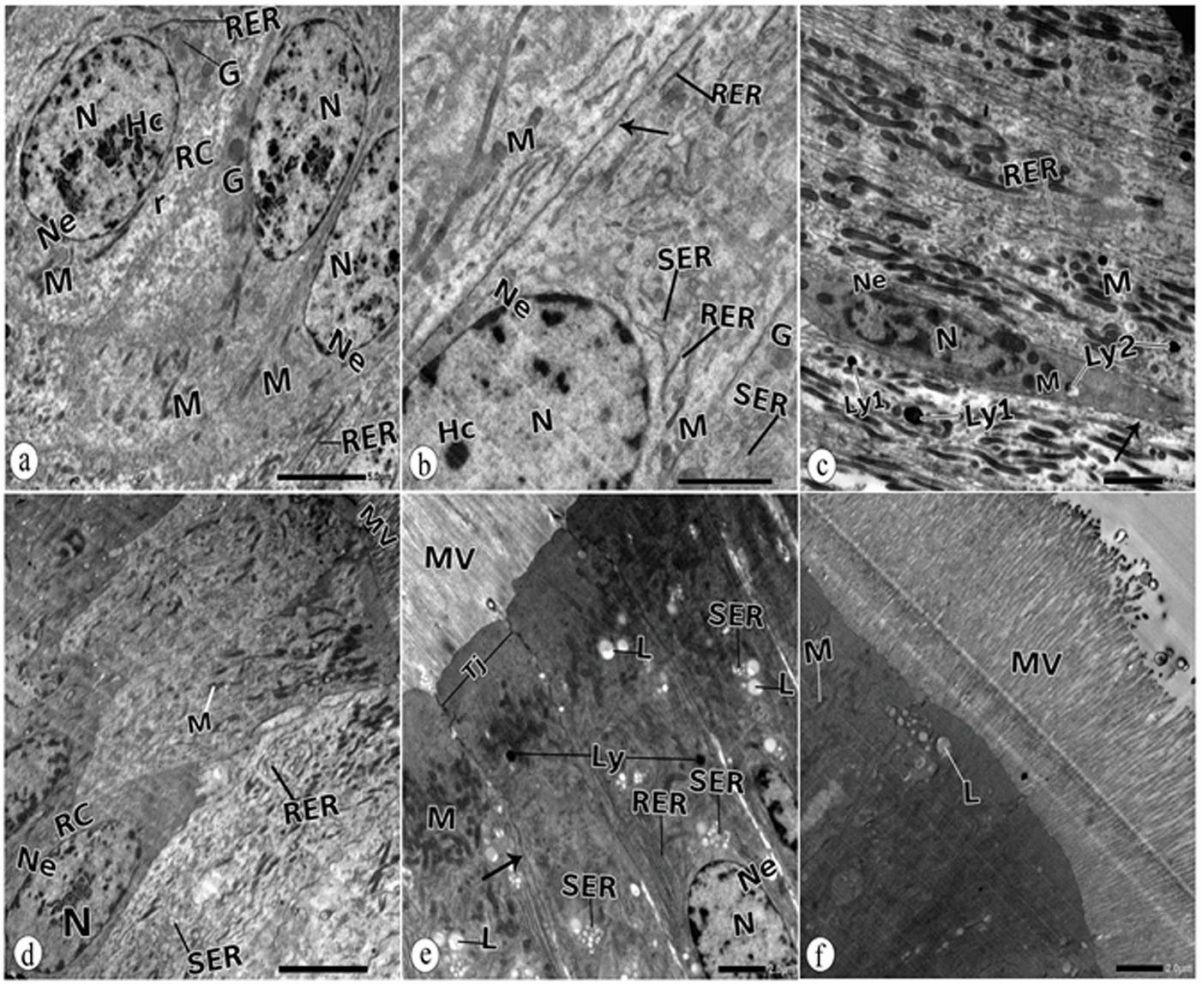
TEM of normal midgut epithelium a: columnar cell (CC) with oval nucleus (N), nuclear envelope (Ne), heterochromatin (Hc), mitochondria(M), rough endoplasmic reticulum (RER), free ribosomes (r). b: nucleus (N), nuclear envelope (Ne), mitochondria (M), heterochromatin (Hc), rough endoplasmic reticulum (RER), smooth endoplasmic reticulum (SER), cell boundary (arrow). c: columnar cell with nucleus (N), nuclear envelope (Ne), mitochondria (M), rough endoplasmic reticulum (RER), primary lysosomes (Ly1), secondary lysosomes (Ly2). d: regenerative cell (RC), nucleus (N), nuclear envelope (Ne), mitochondria(M), rough endoplasmic reticulum (RER), smooth endoplasmic reticulum (SER), microvilli (MV). e: basal nucleus (N), nuclear envelope (Ne), apical mitochondria(M), rough endoplasmic reticulum (RER), smooth endoplasmic reticulum(SER), lysosomes (L), lipid vacuole (L), cell boundary (arrow), tight junction (Tj), microvilli (V). f: apical mitochondria (M), lipid vacuole (L), brush border with long microvilli (MV).

Electron micrographs of the midgut of beetles in the untreated group revealed that the regenerative and columnar cells exhibited oval nuclei with patches of heterochromatin and defined nuclear envelopes (Fig 7A and 7D). Cell boundaries and tight junctions were obvious (Fig 7B). Mitochondria, numerous cisterns of the rough endoplasmic reticulum, and free ribosomes were uniformly distributed in the cytoplasm (Fig 7A–7D). Frequent mitochondria in the basal region were noticed (Fig 7C–7F). Unalterable distributed microvilli cover the luminal border (Fig 7D and 7E). Few lysosomes and lipid vacuoles were distinguished (Fig 7C, 7E and 7F).

### 3.9. Ultrastructure observations of midgut tissues of adult *B*. *polychresta* in the treated group

**Fig 8:**
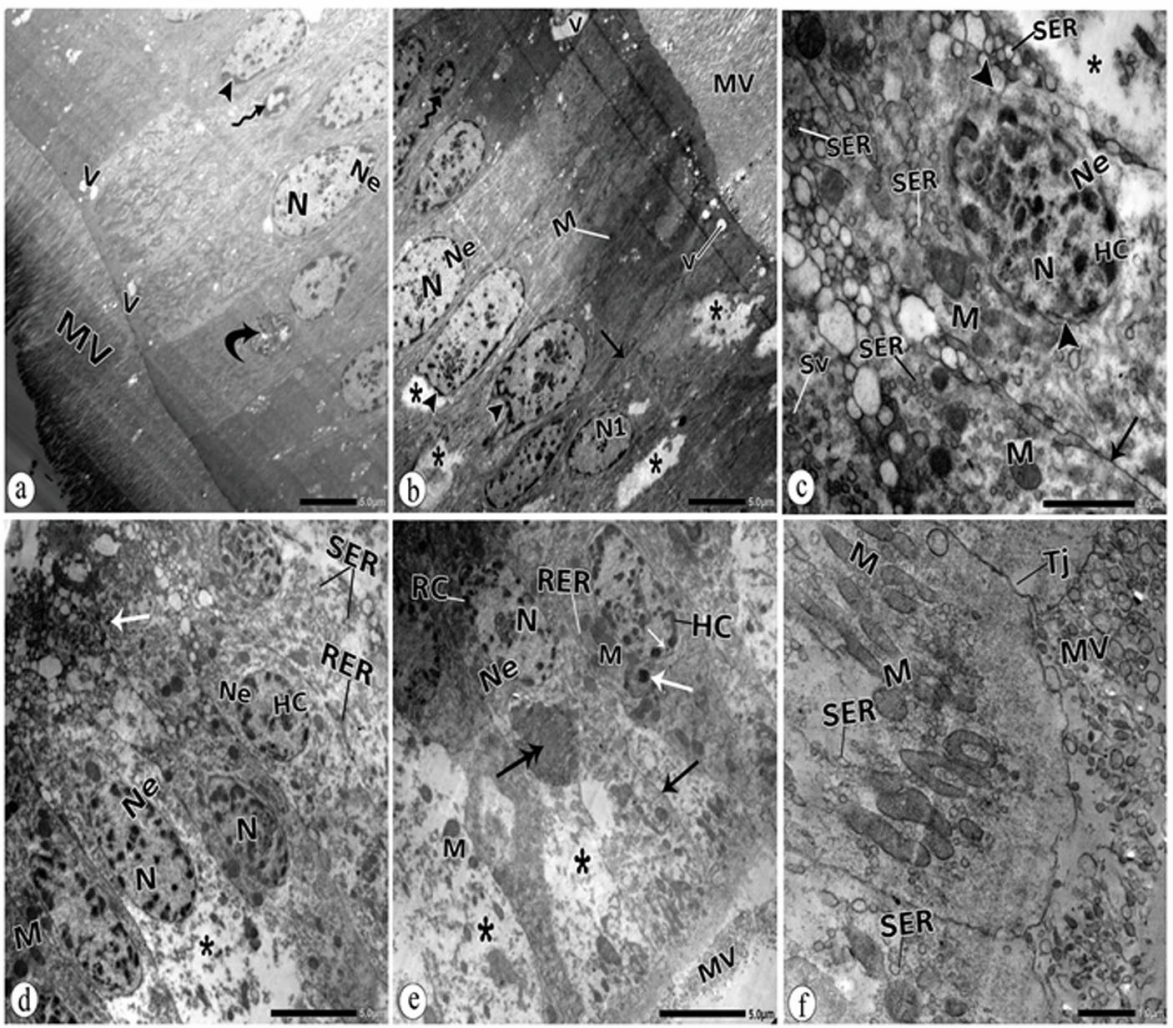
TEM of the treated midgut epithelium a: nucleus (N) with irregular nuclear envelope Ne (head arrow), pyknotic nucleus (wavy arrow), vacuoles (V), myelin figure (curved arrow), microvilli (MV). b: lytic cytoplasm (*), intended nuclear envelopes (head arrow), early apoptotic nucleus (wavy arrow), achromatic nucleus (N1), vacuoles (V), microvilli (MV). N: nucleus, Ne: nuclear envelope. c: lytic cytoplasm (*) heterochromatic nucleus (N), heterochromatin (Hc), karyorrhexis (head arrow) at the nuclear envelope (Ne), swollen mitochondria (M), dilated smooth endoplasmic reticulum (SER), secretory vesicle (Sv). d: lytic cytoplasm (*), nucleus (N), heterochromatin (Hc), nuclear envelope (Ne), electron-dense particles (white arrow), mitochondria (M), dilated smooth endoplasmic reticulum (SER), rough endoplasmic reticulum (RER). e: lytic cytoplasm (*), electron-dense particles (white arrow), heterochromatin (Hc), irregular nuclear envelope Ne (head arrow), swollen mitochondria (M), aggregation of malformed mitochondria (double head arrow), ruptured cell boundary (arrow), distorted microvilli (MV). f: brush border with distorted microvilli (MV), swollen mitochondria (M), dilated smooth endoplasmic reticulum (SER), tight junction (Tj).

Alterations in the midgut cells were observed in the treated beetles. Some nuclei exhibited indentation of the nuclear membranes and pyknotic ones were visible (Fig 8A and 8B). An early apoptotic and achromatic nucleus was detected (Fig 8B). Besides, karyorrhexis was noticed (Fig 8C). The cytoplasm appeared with frequent vacuolations and lytic areas (Fig 8A–8D). Myelin figures were also detected (Fig 8A). Spherical electron-dense particles composed of nanoparticles were distinguished (Fig 8D and 8E). Mitochondria appeared swollen and malformed (Fig 8E). Dilated smooth endoplasmic reticulum (Fig 8C, 8D and 8F), distortion in the brush border of the microvilli (Fig 8E and 8F), and rupture of the cell boundary were observed (Fig 8E).

## 4. Discussion

Because of the growing interest in using NPs in industry, they are being released into the environment [41] NPs can combine with contaminants due to their high surface to mass ratio, which is a path for ecotoxicity [42]. This combination depends on the physical and chemical properties of the NPs [43]. The liberation of NPs into the soil or water could become a potential lethal factor that induces cellular toxicity [44]. Ni-NPs toxicity is yet to be completely explained. In the present study, NiO-NPs cytotoxicity has been illustrated in the ground beetle *B*. *ploycresta* as a biological model. Because beetles can settle in contaminated habitats, they have been successful in biomonitoring programs [10, 17, 32]. Nanoparticles can enter organisms through ingestion or inhalation and are partitioned into tissues where they exert toxic effects [45]. In the current study, a unique route of administration of serial doses of NiO-NPs to the beetles through injection was obtained. This administration route confirms that the chosen doses enter the insect’s body. A previous study by Magaye *et al*. [9] reported that the intravenous injection of metallic Ni-NPS in rats caused severe lung and liver injuries.

In the study, Ni was detected by X-ray microanalysis in the midgut tissues of the treated beetles. X-ray microanalysis is a valid tool for illustrating the distribution of metals in biological tissues [46]. The small size, great capacity, and high reactivity of NPs facilitate their transportation to cellular organelles and exert harmful effects, unusual in their micron-sized counterparts [44]. Little data is available in the literature about the toxicity of Ni-NPs in biological tissues. It was reported by Capasso *et al*. [47] that NiO-NPs induced cell cycle retardation in cell lines of human pulmonary epithelials (BEAS-2B and A549). High doses of oral administration of NiO-NPs caused chromosomal aberrations and DNA breaks in Wistar rats [48]. Recent studies show that NiO-NPs have a genotoxic and mutagenic effect in *Drosophila* [6, 49]. Also, Magaye *et al*. [50] stated that Ni-NPs can initiate intracellular oxidative stress, damaging DNA in cells, which is accountable for their genotoxicity.

Toxicological endpoints can be obtained from mortality tests [51]. The mortality test in the present study revealed that the 0.06 mg/g dose resulted in 100% mortality, while the 0.04 mg/g resulted in 50% mortality. Thus, the selected sublethal dose was 0.02 mg/g. Dabour *et al*. [31] reported the mortality of honey bee workers (*Apis melifera*) due to exposure to sublethal concentrations of PbO and CdO NPs (0.65 mg/ml, and 0.01 mg/ml respectively), which is hardly comparable to the present work.

In the present study, a disturbance in the enzyme activities (significant elevation in AST, ALT and significant inhibition of APOX) was reported in the treated group compared with the untreated one. The elevation in transaminase activities might be required to shift amino acids to the tricyclic acid cycle, so they can be used as fuel molecules to produce additional energy in the stressed organism [52]. Transaminase activities were remoulded during various pathological conditions [53, 54]. This insinuates that injected NiO-NPs may generate toxic reactions. Upadhyay [52] reported that the elevation in the activities of AST and ALT designates tissue damage. After fourteen days of intravenous injection, Magaye *et al*. [9] discovered that Ni nanoparticles significantly increase alkaline phosphatase (ALP) and significantly decrease alanine aminotransferase (ALT) in the liver of rats, while no significant difference in aspartate aminotransferase (AST) is observed. Stohs *et al*. [55] stated that the molecular action of heavy metals provokes ROS, which ushers in cell toxicity. Ni-NPs were found to induce oxidative stress evinced by the generation of reactive oxygen species (ROS), which suggests that the Ni-NPs are capable of inducing genotoxic effects [6, 56]. Some research has proved that diverse kinds of nanoparticles can trigger oxidative stress in arthropod tissues [57, 58]. Rai *et al*. (2014) [59] stated that the penetration of the nanoparticles through the exoskeleton can induce toxicity. Nanomaterials bind to sulphur from proteins or to phosphorus from DNA in the intracellular space, leading to denaturation of enzymes and organelles [60]. Nel *et al*. (2006) [61] and Wise *et al*. [62] stated that oxidative stress and ROS generation are possible mechanisms of cytotoxicity related to NP exposure. Moreover, it has been suggested that NPs induce oxidative stress that leads to DNA damage and apoptosis [63]. Siddiqui et al. (2012) [64] found that NiO-NPs increased apoptosis in MCF-7 and HEp-2 cells *in vitro*. The inhibition of APOX activities in the study might be an outcome of structural alterations of proteins, damage, and finally deactivation of the enzymes [65–67]. APOX catalysed the reduction of H_2_O_2_ using ascorbate as a reducing agent. Therefore, the activity of the enzyme can be limited by the prevalence of reduced ascorbate. The production of reduced ascorbate is sustained under favourable conditions, although the process could be disrupted under stressed conditions [67]. Lijun *et al*. 2005 [68] stated that antioxidant enzymes activities, such as APOX are changed at high Cd concentrations as an outcome of protein alteration. Due to the shape and surface characteristics charge of NPs, they can bind to the proteins and generate adverse biological outcomes such as protein unfolding, thiol crosslinking, fibrillation, and loss of enzymatic activity [69]. Also, cytotoxicity will occur from the release of toxic ions because the thermostatic properties of materials favour particle cessation in a biological medium [69]. However, in the light of these conclusions, NiO-NPs might have a role in enzymatic activity alterations and protein denaturation.

The histological observations of the midgut of the NiO-NPs treated group showed acute and irreversible pathological anomalies. Abdollahi *et al*. [70] stated that the toxicity of xenobiotics is related to the production of free radicals, which are implicated in physiological and histological pathology. A disorganisation of the microvilli was noticed in our preparations that may be a consequence of the rupture of the peritrophic membrane. In the midgut of insects, the peritrophic membrane envelopes the food [71]. Abu El-Saad *et al*. [72] reported that the midgut epithelium is the main object of toxins as it is considered the first barrier against the intoxication of the organism. Other observed alterations in the current study included disruption of the epithelial cells as observed by Rawi *et al*. [73], and the appearance of vacuoles as reported by Younes *et al*. [74], Adel *et al*. [75] and Osman *et al*. [34]. These pathological alterations in the cells may affect the normal physiology of insects [76]. It was cited that zinc oxide nanoparticles led to several morphological and histological abnormalities in *Ae*. *aegypti* third instar larvae (exposed to LC_50_ of 1.57 mg/l for 24 h), including shrinkage in the abdominal region, thorax shape changes, and midgut damage [77].

Several types of research have postulated the histopathological effects of metal/metalloids on the insect’s gut. Zhang *et al*. [29] observed stretching of the cellular axis, an increase of the cellular volume, cytoplasmic vacuolations, and an inhibition of basophilic secretions towards the lumen in the midgut of *Blattella germanica* after treatment with heavy metals (Hg, Pb and Cr). Al-Dhafar & Sharaby [78] observed degeneration, vacuolation, and shrinkage of some epithelial and goblet cells in the midgut of the larvae of *Rhynchophorus ferrugineus* after treatment with ZnSO4. Cid *et al*. [79] observed histological anomalies (vacuolisation and thickening) in digestive gland cells of freshwater bivalves (*Corbicula fluminea*) after exposure to various concentrations of nanodiamonds (NDs, 0.01, 0.1, 1, and 10 mg/l) for 14 days.

Krishnan *et al*. [80] attributed the damage to the gut to the ROS effect. An excess of ROS reduces nutrient absorption and damages midgut cells, resulting in a nonfunctional digestive system. According to Krishnan and Kodrik [81], ROS can oxidise PUFA in the cell membranes and inhibit its function. Dabour *et al*. postulated the adverse consequences of food administration of sublethal concentrations of CdO and PbO NPs on the cellular and subcellular structures of the midgut of honey bee workers (*Apis mellifera*). They found that CdO and PbO NPs marked out various histological anomalies in the NPs fed group compared with controls. One of the histopathological alterations in the midgut tissues of the NiO-NPs treated group in the current study is the rupture of PM. These results are consistent with those reported by Dabour *et al*. [31] who noticed the destruction of PM after food administration of sublethal concentrations of CdO and PbO to honey bee workers (*Apis mellifera*). The PM serves as a protective barrier against chemical, physical and microbial food components [82, 83]. Therefore, any alteration in its structure leads to deleterious effects on the midgut tissues of insects.

Ultrastructure deformities in the NiO-NPs treated group involve chromatin clumping and the presence of pyknotic nuclei, which suggests less efficient transcription and subsequently result in the decrease in metabolic activity [84]. This also indicates that cells are in an advanced cell death process [85, 86]. The irregulation of the nuclear envelopes and karyorrhexis indicate a pathway of cell death [87, 88]. Some authors attributed apoptosis to metal accumulation [89, 90], which is in agreement with our results. Research has proven the relation between exposure to metals and the prevalence of apoptosis [91, 92]. The vacuolated areas in the cytoplasm may be ascribed to the action of lysosomal hydrolase or the breakage of the mitochondria [93] and sometimes may be due to the enhanced endocytotic activity as described by Cavados *et al*. [94].

Nanoparticles are frequently distinguished from lysosomes upon internalisation, and numerous nanomaterials have been related to lysosomal impairments [95]. It has been confirmed that lysosomal destabilisation activates mitochondrial apoptosis [96, 97].

The study revealed the presence of electron-dense particles in the midgut tissues due to NiO-NPs accumulations as a detoxification mechanism [98, 99]. Our results follow Polidori *et al*. (2018) [93], who detected metal precipitation in spherites in the midgut of paper wasps (*Polistes dominula*) collected from urban environments. Also, a similar observation was noticed by Pigino *et al*. [100], who reported that a large number of electron-dense granules, composed of a variety of heavy metals, were accumulated in the epithelium of the midgut ventriculus of the mite *Xenillus tegeocranus* from a deserted mining and smelting area. Karpeta-Kaczmarek *et al*. [101] reported that the epithelial cells of midgut and hindgut of *Acheta domesticus* (Orthoptera, Gryllidae) were damaged at high concentrations of nanodiamonds (NDs) and autophagy was activated. The hydrophobic nature of NPs allows them to cross the cell membranes [102] and then they may act as centres of oxidative damage inside the cell [103].

Disruption of microvilli is one of our major findings in this study since it is the first site facing and interacting with pollutants [93]. Some changes in the cytoplasmic organelles were distinguished in our electron micrographs, such as lysis of mitochondrial matrices, dilated rough and smooth endoplasmic reticulum, and the presence of myelin figures. It was found that heavy metals distort cytoplasmic membranes [10, 17]. Mitochondrial alteration is a reflection of the deregulation of mitochondrial membrane transport [104]. Toxins cause damage to mitochondrial membranes and cristae, as discovered by Braeckman *et al*. [105] in insects’ intoxicated cells. Moreover, the swelling of the mitochondria reflects the entry of water and/or solutes into the mitochondrial matrix [106]. Belyaeva *et al*. [107] deduced that mitochondria are an important target for the toxic effects of metals and their oxide NPs.

The interference of heavy metals with proper processing in the ER causes its dilation and activates the ER stress response [108]. The proliferation of myelin figures in our preparations has been interpreted as a symptom of intoxication trigged by NiO-NPs that implies an adaptive mechanism in response to the high degradation of cellular organelles [109]. These ultrastructure alterations represent the major features of both cell necrosis and apoptosis [110, 111]. In accordance with our results, Dabour *et al*. [31] observed ultrastructure anomalies in the midgut cells of workers of honey bees (*Apis mellifera*) treated with CdO and PbO-NPs.

The present results are considered the first record to present the physiological and histological alterations induced by NiO-NPs in the ground beetle *B*. *polychresta*. However, the processes behind NiO-NPs’ toxicity remain unknown. Additionally, the effect of nanoscale dimensions, form, and charge on the different possible mechanisms of action will be examined in more detail in our upcoming work.

## 5. Conclusion

The findings of the current study will guide researchers to identify the impact of the sublethal dose of NiO-NPs on biological tissues. It could be concluded that particles, which are less than 50 nm, are capable of entering cells and attaching to macromolecules, leading to DNA damage. Thus, precautions should be taken when dealing with minute particles. The mechanisms responsible for the toxicity of NiO-NPs still need to be investigated. Also, the impact of nano size, shape, and charge on the various potential mechanisms of action must be elucidated. Lastly, further efforts are still necessary to validate the proposed metal oxide nanoparticles in field conditions, monitoring at the same time their stability, fate in the environment, and sublethal effects on non-target organisms.

## Supporting information

S1 Fig Photograph of photograph of *Blaps polycresta*. (DOCX)

S2 Fig A photograph shows the route of administration of NiO-NPs ventro-caudal through the arthrodial membrane between the 4^th^ and 5^th^ abdominal sclerites. (DOCX)

S3 Fig Linear dose-mortality response percentages of NiO-NPs in the studied groups. (DOCX)

S4 Fig Energy-dispersive X-ray spectra reveal the qualitative elemental composition as measured in counts per second in the midgut tissues of *B*. *polychresta* of the studied groups. Horizontal scale, X-ray energy; vertical scale, X-ray counts.
(DOCX)

S1 Table Mortality counts of beetles in the untreated and NiO-NPs treated groups for 30 days. (DOCX)

S2 Table Mean± SE of the cumulative mortality percentages in the studied groups. (DOCX)

S3 Table Enzyme activities in midgut tissues (mU/mg protein) of *B*. *polychresta* from the studied groups. (DOCX)
