# Supplementary figures and images for: Biochemical and histological alterations induced by nickel oxide nanoparticles in the ground beetle Blaps polychresta (Forskl, 1775) (Coleoptera: Tenebrionidae)
Source: PLoS One. 2021 Sep 24;16(9):e0255623. doi: 10.1371/journal.pone.0255623 (PMC8462711; doi:10.1371/journal.pone.0255623)

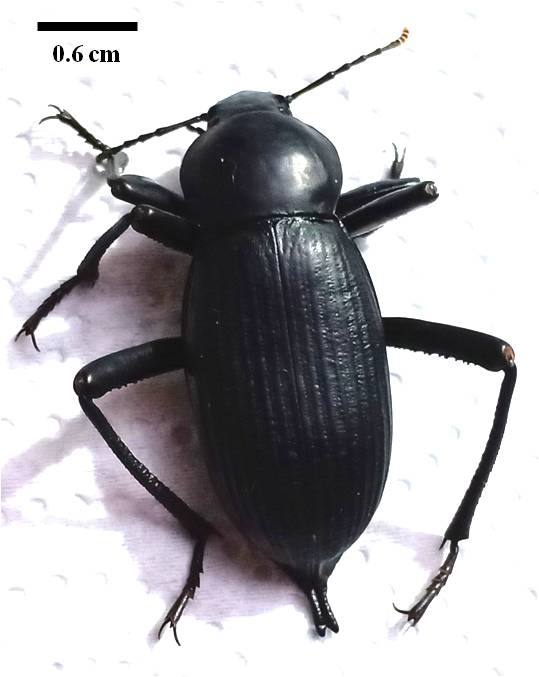


**Fig. S1**

Supplement: S1 Fig — (DOCX) [file pone.0255623.s001.docx]

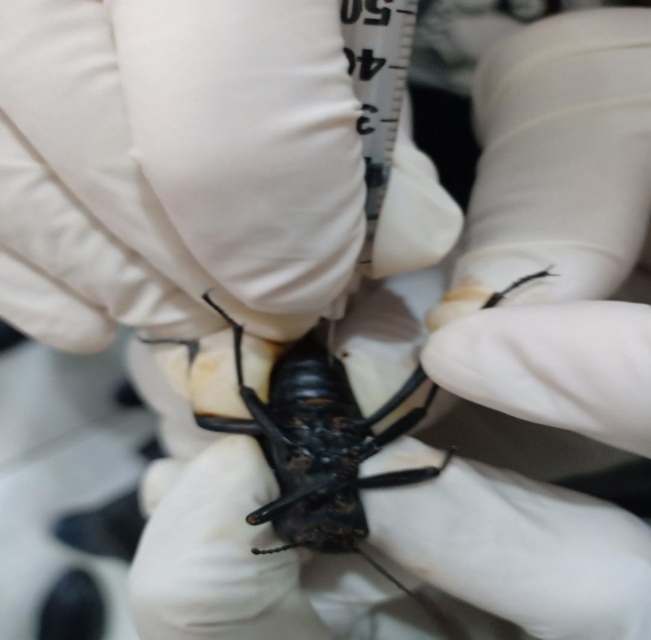


**Fig. S2**

Supplement: S2 Fig — (DOCX) [file pone.0255623.s002.docx]

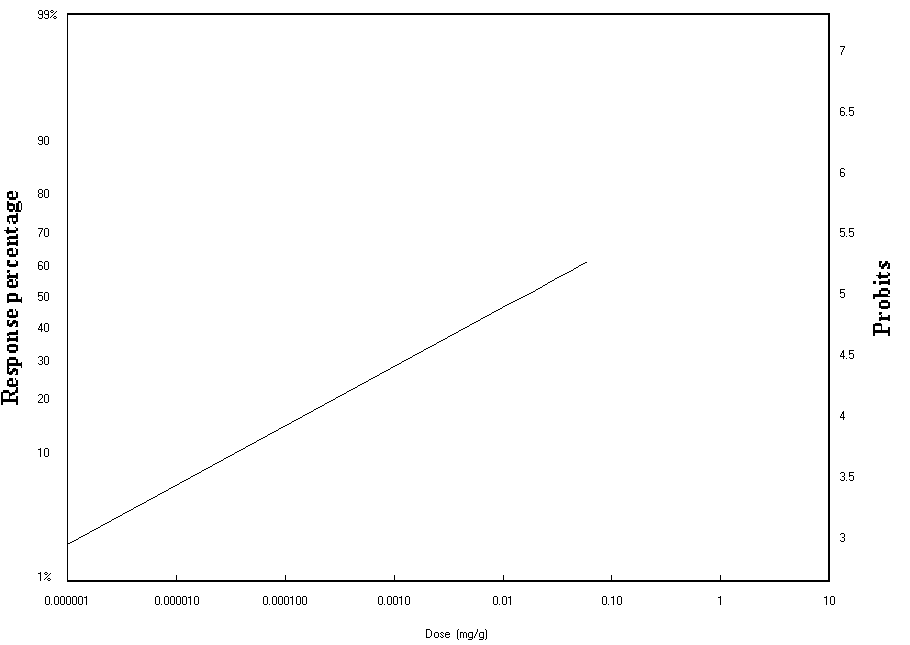


**Fig. S3**

Supplement: S3 Fig — (DOCX) [file pone.0255623.s003.docx]
