# Supplementary material for: Biochemical and histological alterations induced by nickel oxide nanoparticles in the ground beetle Blaps polychresta (Forskl, 1775) (Coleoptera: Tenebrionidae)
Source: PLoS One. 2021 Sep 24;16(9):e0255623. doi: 10.1371/journal.pone.0255623 (PMC8462711; doi:10.1371/journal.pone.0255623)
Supplement: S4 Fig — Horizontal scale, X-ray energy; vertical scale, X-ray counts. (DOCX) [file pone.0255623.s004.docx]

| 0  5  10  15  20  Energy (keV)  0  2  4  6  8  10  Cps  Na  Al  P  S (a) Midgut of *B. polycresta*  Untreated Group      K  Ca  Cu  Zn | 0  5  10  15  20  Energy (keV)  0  2  4  6  8  10  12  Cps  Treated Group (2)  S    P (b) Midgut of *B. ploycresta*  Al  K    K  Na K  Ca  Ca  Ni  Cu  Zn |
| --- | --- |
|  |  |

**Fig. S4**
