# Supplementary material for: Biochemical and histological alterations induced by nickel oxide nanoparticles in the ground beetle Blaps polychresta (Forskl, 1775) (Coleoptera: Tenebrionidae)
Source: PLoS One. 2021 Sep 24;16(9):e0255623. doi: 10.1371/journal.pone.0255623 (PMC8462711; doi:10.1371/journal.pone.0255623)
Supplement: S1 Table — (DOCX) [file pone.0255623.s005.docx]

**Table S1**

| **Mortality period** | **Untreated group**  **n=20** | **Treated groups** | | | | |  |
| --- | --- | --- | --- | --- | --- | --- | --- |
|  |  | **Group1**  **n=20 (0.01 mg/g)** | **Group 2**  **n=20 (0.02 mg/g)** | **Group 3**  **n=20 (0.03 mg/g)** | **Group 4**  **n=20 (0.04 mg/g)** | **Group 5**  **n=20 (0.05 mg/g)** | **Group 6**  **n=20 (0.06 mg/g)** |
| **Day 1** | - | - | - | - | - | 4 | 7 |
| **Day 2** | - | - | - | - | 7 | - | - |
| **Day 3** | - | - | - | 3 | - | - | 1 |
| **Day 4** | - | - | - | - | - | - | - |
| **Day 5** | - | - | 3 | - | - | - | - |
| **Day 6** | - | - | - | - | - | 2 | 1 |
| **Day 7** | - | - | - | - | - | - | - |
| **Day 8** | - | - | - | 4 | 2 | - | - |
| **Day 9** | - | - | - | - | - | - | - |
| **Day 10** | - | - | 4 | - | - | - | 4 |
| **Day 11** | - | - | - | - | - | - | - |
| **Day 12** | - | - | - | - | - | 2 | - |
| **Day 13** | - | - | - | - | - | - | - |
| **Day 14** | - | - | - | - | - | - | - |
| **Day 15** | - | - | - | - | - | 1 | 2 |
| **Day 16** | - | - | - | - | - | - | - |
| **Day 17** | - | 3 | - | - | - | - | - |
| **Day 18** | - | - | - | - | - | - | - |
| **Day 19** | - | - | - | - | - | 1 | - |
| **Day 20** | - | - | - | - | - | - | - |
| **Day 21** | - | - | - | 2 | - |  | 2 |
| **Day 22** | - | - | - | - | 1 | - | - |
| **Day 23** | - | 4 | - | - | - | - | - |
| **Day 24** | 1 | - | - | - | - | 3 | 2 |
| **Day 25** | - | - | - | - | - | - | - |
| **Day 26** | - | - | - | - | - | 1 | 1 |
| **Day 27** | - | - | - | - | - | - | - |
| **Day 28** | - | - | - | - | - | - | - |
| **Day 29** | - | - | - | - | - | - | - |
| **Day 30** | - | - | - | - | - | - | - |
| **Survived beetles** | 19 | 13 | 13 | 11 | 10 | 6 | 0 |
