# Supplementary material for: Biochemical and histological alterations induced by nickel oxide nanoparticles in the ground beetle Blaps polychresta (Forskl, 1775) (Coleoptera: Tenebrionidae)
Source: PLoS One. 2021 Sep 24;16(9):e0255623. doi: 10.1371/journal.pone.0255623 (PMC8462711; doi:10.1371/journal.pone.0255623)
Supplement: S2 Table — (DOCX) [file pone.0255623.s006.docx]

**Table S2**

| **Mortality period** | **Untreated**  **Group n=20** | **Treated groups** | | | | | |
| --- | --- | --- | --- | --- | --- | --- | --- |
|  |  | **Group1**  **n=20 (0.01 mg/g)** | **Group2**  **n=20 (0.02 mg/g)** | **Group3**  **n=20 (0.03 mg/g)** | **Group4**  **n=20 (0.04 mg/g)** | **Group5**  **n=20 (0.05 mg/g)** | **Group 6**  **n=20 (0.06 mg/g)** |
| **Mean ± SE.** | 1.17±0.39 | 12.33±2.75 | 27.0±2.39 | 32.67±2.47 | 43.0±1.77 | 43.67±3.13 | 69.67±4.06 |
| **H(p)** | 140.778^*^(<0.001^*^) | | | | | | |
| ***P* _Untreated_** |  | 0.132 | <0.001^*^ | <0.001^*^ | <0.001^*^ | <0.001^*^ | <0.001^*^ |
| **Significant between groups** |  | p_1_=0.027^*^, p_2_<0.001^*^, p_3_<0.001^*^, p_4_<0.001^*^, p_5_<0.001^*^, p_6_=0.172, p_7_<0.001^*^, p_8_=0.002^*^, p_9_<0.001^*^, p_10_=0.016^*^, p_11_=0.087,p_12_<0.001^*^,p_13_=0.482, p_14_=0.028^*^,p_15_=0.004^*^ | | | | | |

H: H for **Kruskal Wallis test,** Pairwise comparison between each 2 groups was done using **Post Hoc Test (Dunn's for multiple comparisons test).** p: p value for comparing between the studied groups. *: Statistically significant at *p* ≤ 0.05.

**p _untreated_:** p value for comparing between **Untreated group** and each other group

**p_1_:** *p* value for comparing between **Group1** and **Group2**

**p_2_:** *p* value for comparing between **Group1** and **Group3**

**p_3_:** *p* value for comparing between **Group1** and **Group4**

**p_4_:** *p* value for comparing between **Group1** and **Group5**

**p_5_:** *p* value for comparing between **Group1** and **Group6**

**p_6_:** *p* value for comparing between **Group2** and **Group3**

**p_7_:** *p* value for comparing between **Group2** and **Group4**

**p_8_:** *p* value for comparing between **Group2** and **Group5**

**p_9_:** *p* value for comparing between **Group2** and **Group6**

**p_10_:** *p* value for comparing between **Group3** and **Group4**

**p_11_:** *p* value for comparing between **Group3** and **Group5**

**p_12_:** *p* value for comparing between **Group3** and **Group6**

**p_13_:** *p* value for comparing between **Group4** and **Group5**

**p_14_:** *p* value for comparing between **Group4** and **Group6**

**p_15_:** *p* value for comparing between **Group5** and **Group6**
