# Supplementary material for: Biochemical and histological alterations induced by nickel oxide nanoparticles in the ground beetle Blaps polychresta (Forskl, 1775) (Coleoptera: Tenebrionidae)
Source: PLoS One. 2021 Sep 24;16(9):e0255623. doi: 10.1371/journal.pone.0255623 (PMC8462711; doi:10.1371/journal.pone.0255623)
Supplement: S3 Table — (DOCX) [file pone.0255623.s007.docx]

**Table S3**

| **Enzymes** | **Untreated Group** | **Treated group (Group 2)** | **T** | ***P*** |
| --- | --- | --- | --- | --- |
| **AST** | 117.80 ± 3.22 | 211.0 ± 4.96 | 15.767^*^ | <0.001^*^ |
| **ALT** | 57.80 ± 2.67 | 123.40 ± 3.85 | 13.986^*^ | <0.001^*^ |
| **APOX** | 4.76 ± 0.17 | 2.28 ± 0.14 | 11.091^*^ | <0.001^*^ |

t: Student t-test, *p*: *p* value for comparing between the studied groups, *: Statistically significant at *p* ≤ 0.05. Data expressed using mean ± SE (n=8).
